# Virtual multiple errands test (VMET): a virtual reality-based tool to detect early executive functions deficit in Parkinson’s disease

**DOI:** 10.3389/fnbeh.2014.00405

**Published:** 2014-12-05

**Authors:** Pietro Cipresso, Giovanni Albani, Silvia Serino, Elisa Pedroli, Federica Pallavicini, Alessandro Mauro, Giuseppe Riva

**Affiliations:** ^1^Applied Technology for Neuro-Psychology Lab, IRCCS Istituto Auxologico ItalianoMilano, Italy; ^2^Division of Neurology and Neurorehabilitation, IRCCS Istituto Auxologico ItalianoOggebbio, Italy; ^3^Department of Psychology, Università Cattolica del Sacro CuoreMilano, Italy

**Keywords:** virtual reality, executive function, VMET, psychometric assessment, Parkinson’s disease, mild cognitive impairment

## Abstract

**Introduction**: Several recent studies have pointed out that early impairment of executive functions (EFs) in Parkinson’s Disease (PD) may be a crucial marker to detect patients at risk for developing dementia. The main objective of this study was to compare the performances of PD patients with mild cognitive impairment (PD-MCI) with PD patients with normal cognition (PD-NC) and a control group (CG) using a traditional assessment of EFs and the Virtual Multiple Errands Test (VMET), a virtual reality (VR)-based tool. In order to understand which subcomponents of EFs are early impaired, this experimental study aimed to investigate specifically which instrument best discriminates among these three groups.

**Materials and methods**: The study included three groups of 15 individuals each (for a total of 45 participants): 15 PD-NC; 15 PD-MCI, and 15 cognitively healthy individuals (CG). To assess the global neuropsychological functioning and the EFs, several tests (including the Mini Mental State Examination (MMSE), Clock Drawing Test, and Tower of London test) were administered to the participants. The VMET was used for a more ecologically valid neuropsychological evaluation of EFs.

**Results**: Findings revealed significant differences in the VMET scores between the PD-NC patients vs. the controls. In particular, patients made more errors in the tasks of the VMET, and showed a poorer ability to use effective strategies to complete the tasks. This VMET result seems to be more sensitive in the early detection of executive deficits because these two groups did not differ in the traditional assessment of EFs (neuropsychological battery).

**Conclusion**: This study offers initial evidence that a more ecologically valid evaluation of EFs is more likely to lead to detection of subtle executive deficits.

## Introduction

The umbrella term “executive function” (EF) refers to a broad set of high-level cognitive abilities used to regulate actions (Burgess and Simons, 2005; Chan et al., 2008; Otero and Barker, 2013). These cognitive abilities range from the capacity to problem solve, plan, sustain attention, utilize internal/external feedback, multitasking and cognitive flexibility and ability to deal with novelty (Damasio, 1995; Stuss et al., 1995; Grafman and Litvan, 1999; Burgess et al., 2000; Miller and Cohen, 2001; Strauss et al., 2006; Stuss, 2007; Chan et al., 2008; Goldberg, 2009). Impairment of EF is extremely common in neurological patients, specifically in those presenting with frontal pathology (Bechara et al., 1994; Stuss et al., 1995; Burgess and Shallice, 1996a,b; Dreher et al., 2008; Barker et al., 2010; Morton and Barker, 2010; Cole et al., 2013). Although EFs are thought to be mediated by frontal brain regions, frontal areas have multiple connections with cortical and subcortical regions, as well as to the amygdala, cerebellum, and basal ganglia (for a review, see Tekin and Cummings, 2002). Specifically, functional magnetic resonance imaging (fMRI) studies have shown that BOLD signals increase in the basal ganglia during the performance of EF tasks which require cognitive flexibility, shifting of mental sets, and updating of working representations (Cools et al., 2004; Leber et al., 2008; Hikosaka and Isoda, 2010). Further evidence that the basal ganglia is part of the circuitry crucial for executive functioning comes from studies with patients with basal ganglia lesions, specifically patients who suffer from Parkinson’s Disease (PD; Cools et al., 1984, 2001; McKinlay et al., 2010). Indeed, in addition to the typical motor signs, a number of different cognitive deficits have received relevant clinical attention in PD (Levy et al., 2002; Vingerhoets et al., 2003; Foltynie et al., 2004; Muslimović et al., 2005; Williams-Gray et al., 2009). The characteristics of cognitive impairment in PD may be extremely variable in regard to the timing of the onset and the rate of progression (Aarsland et al., 2005, 2007; Buter et al., 2008; Hely et al., 2008), and in terms of what cognitive functions are impaired (Verleden et al., 2007; Kehagia et al., 2010). Even if the neuropsychological profile of patients who suffer from PD is heterogeneous, including memory deficits (Whittington et al., 2006; Ramanan and Kumar, 2013) and visuo-spatial impairments (Montse et al., 2001; Kemps et al., 2005), it is marked specifically by executive deficits (Cools et al., 2001; McKinlay et al., 2010). Moreover, the impairment of EFs appears to be the core feature of a neuropsychological profile in PD-related dementia (Girotti et al., 1988; Jacobs et al., 1995; Padovani et al., 2006; Pagonabarraga and Kulisevsky, 2012; Kudlicka et al., 2013). Similar executive deficits also can be found in nondemented PD patients (for reviews, see Kudlicka et al., 2011; Ceravolo et al., 2012), but they are more severe in patients who suffer from dementia. Following this direction, several recent studies have pointed out the predictive value of early EF deficits in the transitional stage of mild cognitive impairment (MCI) of the disease (Levy et al., 2002; Woods and Tröster, 2003). The concept of MCI, originally introduced to identify the earliest cognitive changes due to Alzheimer’s Disease (AD; Petersen et al., 2001; Petersen, 2004), has also been applied to PD to improve the detection of patients at risk for developing dementia (Aarsland et al., 2011). Litvan et al. (2012) proposed the diagnostic guidelines to facilitate the diagnosis of “mild cognitive impairment in Parkinson’s Disease” (PD-MCI). These criteria are generally based on the established principles of MCI given by Petersen, namely, subjective cognitive decline and objective evidence of impairment assessed by neuropsychological evaluation that does not interfere with functional independence (Petersen, 2004). Similar to AD, the risk of developing dementia increases appreciably with the presence of PD-MCI (Janvin et al., 2006). As underlined by Biundo et al. (2013), a great challenge today is to characterize the neuropsychological profile of PD-MCI and to evaluate the screening power of traditional neuropsychological tests. In their work, 104 PD patients were given an extensive neuropsychological evaluation. Results showed that specific neuropsychological tests measuring attentional/set-shifting, verbal memory, and visual-spatial functions are the best predictors of PD-MCI. In this perspective, EF dysfunction is a possible marker of potentially more severe cognitive impairment and may indicate a likely decline into dementia. Similarly, Goldberg proposed that EF deficits are also key markers for later dementia in AD (Goldberg, 2009). Petrova et al. compared the performances of 23 patients suffering from amnestic PD-MCI utilizing 25 cognitively healthy controls to investigate which subcomponent of EFs are impaired in PD-MCI patients (Petrova et al., 2010). The diagnosis of MCI was made according to modified criteria proposed by Petersen et al. (2001). They found that amnestic PD-MCI patients showed impairment in several aspects of attention/EFs, including the ability to inhibit irrelevant responses and in cognitive flexibility, as measured by the Stroop test (Stroop, 1935) and Modified Wisconsin Card Sorting Test (Nelson, 1976), in formulating and following a complex plan, as revealed by Trail Making Test (Greenlief et al., 1985), and in sustaining a cognitive load during a language test, as highlighted by the phonemic and semantic verbal fluency test (Lezak, 1995). These findings underline the need for a complex evaluation of EFs in MCI-PD patients, especially in the possible relationship between these early executive impairments and behavioral change.

Previous studies indicate a need for rigorous ecologically valid assessments that reliably capture subtle impairments that may be markers for later dementia. In fact, there are some critical issues in the traditional neuropsychological evaluation of EFs (Chan et al., 2008). A more ecological and prompt assessment of EFs is essential to evaluate the specific cognitive profile of different individuals (Goldstein, 1996; Chaytor and Schmitter-Edgecombe, 2003; Burgess et al., 2006). Indeed, the traditional evaluation does not reflect the complexity of EFs in everyday situations. A more detailed assessment may evaluate if individuals are able to formulate, store, and check all the goals and subgoals in order to effectively respond to environmental and/or internal demands. In this direction, there are some instruments developed to measure executive deficits in situations similar to daily ones, such as the Behavioral Assessment of Dysexecutive Syndrome (BADS; Wilson et al., 1996) and the Multiple Errands Test (MET; Shallice and Burgess, 1991). The BADS (Wilson et al., 1996) consists of six subtests and a Dysexecutive Questionnaire (DEX). The DEX is designed to assess everyday cognitive, emotional, and behavioral changes, and it is completed by the patient (self-rating: DEX-S) and a person who knows the patient (independent rater: DEX-I). Although the BADS has good validity (Wilson et al., 1998), and the DEX was recently found to be, with some limitations, a useful instrument for capturing changes in to day to day functioning (Barker et al., 2011), it does not measure performance during real-life tasks. An interesting example of a functional instrument is the MET (Shallice and Burgess, 1991), in which participants are invited to complete different tasks following specific rules to adhere to within a specified time frame. Even the simplified versions of the MET, however, adapted especially to be performed in a hospital setting or a nearby shopping mall (Alderman et al., 2003), can be particularly demanding for a patient because these versions require good motor skills; for a clinician these versions are time consuming and demand high economic costs.

To address the issue of ecological validity and clinical utility, virtual reality (VR) appears to be an appropriate instrument for the evaluation of EFs because it provides the chance to deliver different tasks within ecologically valid, controlled, and secure environments (for a review, see Bohil et al., 2011). Based on this, the virtual version of the Multiple Errands Test (VMET) has been recently developed and tested in different clinical populations (Albani et al., 2011; Raspelli et al., 2012; Cipresso et al., 2013a). The VMET is a VR-based tool aimed at evaluating different aspects of EFs by enabling active exploration of a virtual supermarket, where participants are requested to buy various products presented on shelves and to abide by different rules. Thanks to the potential of the VR, with the VMET the real functional status of patients can be easily evaluated, as manifested in executive dysfunctions, which had not been fully acknowledged in laboratory tests. Specifically, the VMET measures a patient’s ability to formulate, store, and check all the goals and subgoals to effectively respond to environmental demands in ecological situations and to complete the specified task. The VMET has demonstrated good inter-rate reliability, showing an intraclass correlation coefficient (ICC) of 0.88 (Cipresso et al., 2013b) and good usability (Pedroli et al., 2013). This test has demonstrated that it can be used with patients who are not familiar with computerized tests. On the basis of these methodolical strengths, we argue that the VMET may significantly improve the traditional assessment of EFs in PD-MCI patients.

The main objective of this study is to compare the performances of PD-MCI with PD with normal cognition and cognitively healthy controls using traditional assessments of EFs and the VMET. In order to understand which subcomponents of EFs are early impaired, this experimental study aimed to specifically investigate the instruments that best discriminate among these three groups.

## Materials and methods

### Participants

A total of 45 participants allocated to three groups were included in the study: 15 PD patients with normal cognition (PD-NC), 15 PD patients suffering from MCI, and 15 cognitively healthy individuals (CG, control group). The PD-NC group was composed of six women (40%) and nine men (60%), while the PD-MCI and the CG included seven women (46.7%) and eight men (53.3%) and nine women (60%) and six men (40%), respectively. CG and PD patients were recruited from the San Giuseppe Hospital’s Istituto Auxologico Italiano in Verbania, Italy. Individuals did not receive money for their participation in the study. Detailed demographic and clinical characteristics of the three groups are reported in Table 1. Individuals gave their written consent for the procedures, which were approved by the Ethical Committee of the Istituto Auxologico Italiano.

**Table 1:** **Demographic characteristics of the three groups of the study: PD patients with normal cognition (PD-NC), PD patients suffering from mild cognitive impairment (PD-MCI), and healthy individuals (CG, control group)**.

Variables	Group		
	PD–NC (*n* = 15)	PD–MCI (*n* = 15)	CG (*n* = 15)
Age	69 (8.1)	68.1 (9.4)	61.7 (5.2)
Years of Education	7.93 (3.7)	7.2 (3.3)	12.2 (3.1)

### Neuropsychological global assessment and Parkinson’s Disease classification

PD patients were classified into the two cognitive groups (PD-NC and PD-MCI), following the guidelines of the Task Force for the diagnosis of PD-MCI (Litvan et al., 2012). The proposed PD-MCI criteria utilized a two-level schema depending on the comprehensiveness of the neuropsychological testing. The Level I and II categories represent PD-MCI, but they differ in regard to the type of neuropsychological assessment and, consequently, the level of diagnostic certainty. Specifically, for the diagnosis of PD-MCI by Level II criteria, the Task Force recommends comprehensive neuropsychological testing that highlights either two impaired tests in one cognitive domain or one impaired test in two different cognitive domains. For the division of PD patients into PD-ND and PD-MCI (Level II), a comprehensive neuropsychological battery with at least two neuropsychological tests per cognitive domain was employed. First, to evaluate the cognitive functioning of the participants in the study, the Mini Mental State Examination (MMSE; Folstein et al., 1975) was administered. The MMSE is a brief questionnaire widely used to obtain a picture of an individual’s present cognitive performance in different cognitive domains (short- and long-term memory, orientation, attention, verbal fluency, and constructional apraxia). A score of <24 is generally the accepted cutoff, indicating the presence of cognitive impairment. The MMSE has been validated in the Italian sample with 1019 elderly subjects (aged 65–89 years) (Magni et al., 1996).

To evaluate the visuo-spatial function, the Behavioral Inattention Test (BIT; Wilson et al., 1987) was used. The BIT is traditionally used to screen for neglect behaviors, and it consists of six conventional pencil and paper subtests and nine behavioral subtests reflecting aspects of daily life. In the present study, the Italian validation of the BIT’s conventional subtests was administered (Wilson et al., 2010): line crossing, letter cancellation, star cancellation, figure and shape copying, line bisection, and representational drawing. The maximum total score is 146 points.

To assess language comprehension abilities, the Token test was administered within the brief neuropsychological examination (Mondini et al., 2003). This is a simple test which requires 20 tokens that vary in shape, color, and size. The Italian validated test has 32 commands, each of which requires the attention and/or the manipulation of one or more of the tokens (e.g., “Put the small red square under the white large circle.”).

The Italian validated Digit Span was used to evaluate short-term memory abilities (Orsini et al., 1987). In this easy-to-administer test, the researcher reads a series of digits aloud to the participant, who is requested to repeat back the same series of digits in the same sequence (i.e., 9–1–7 for 9–1–7). To assess long-term memory abilities, the Short Story test (Novelli et al., 1986a) was administered. The researcher read aloud the Short Story, required participants to provide a first immediate recall, then read aloud the story again, requesting another immediate recall. After a delay of around 15 min, participants were asked for a delayed retrieval. The final score is the average of the number of correctly recalled morphological units over three recall trials.

In order to specifically evaluate the spatial memory abilities of the study’s participants, the following standard neuropsychological test was administered: the Corsi Block Test (Corsi, Unpublished Thesis; Spinnler and Tognoni, 1987). This task is used to measure short-term spatial memory (Corsi Span) and long-term spatial memory (Corsi Supraspan). The participants are invited to tap a sequence of wooden blocks in the same order as the researcher, with increasing span length on each trial.

Neuropsychological data for the three groups are reported in Table 2.

**Table 2:** **Mean scores of neuropsychological global assessment tasks reported by the three groups of the study**.

Variables	Group		
	PD–NC (*n* = 15)	PD–MCI (*n* = 15)	CG (*n* = 15)
MMSE	27 (1.8)	25.1 (3.2)	29.7 (1.0)
BIT	140 (9.6)	130 (13.3)	144 (1.3)
Token test	33.1 (2.1)	30.1 (2.1)	33.9 (1.0)
Digit span	5.5 (1.2)	5.1 (1.2)	6.2 (0.9)
Corsi block test—span	4.61 (0.8)	3.4 (1.3)	5.5 (0.9)
Short Story	14 (2.8)	10.3 (3.0)	14.8 (4.3)

All scores obtained from these neuropsychological tests have been corrected for age, education level, and gender, according to Italian normative data.

### Executive functions evaluation

In order to fully evaluate the executive functioning of the study participants, a comprehensive standard neuropsychological battery focused on the different aspects of EF was administered.

The Clock Drawing Test (Freedman et al., 1994; Caffarra et al., 2011) has been traditionally used to assess a wide range of cognitive abilities including EFs, specifically understanding verbal instructions and abstract thinking and planning abilities. It is brief, easy to administer, and has excellent patient acceptability. Participants were required to draw numbers in a circle on a paper to resemble a clock and then draw the hands of the clock to read “10 after 11”.

To evaluate multi-tasking and cognitive flexibility, two types of verbal fluency tests were employed. Phonological verbal fluency (Novelli et al., 1986b; Lezak, 1995) is a traditional neuropsychological measure of language production in which a number of words are given with an initial letter (e.g., F). Semantic verbal fluency (Novelli et al., 1986b; Lezak, 1995) is a more complex traditional neuropsychological measure of language production in which the number of words in a specific category produced in 60 s (e.g., animals) is evaluated. Both tests require participants to use executive processes to solve them because an efficient and creative organization of the verbal retrieved material, as well as the inhibition of responses when appropriate, is crucial.

To specifically detect early deficits in problem-solving and planning, the Tower of London test (Shallice, 1982; Fancello et al., 2006) was administered. The researcher explained the rules of the task (e.g., don’t make more moves than necessary), and then used one tower with three rods of descending heights and a set of beads to display the desired goal: Participants are invited to rearrange the set of beads on the tower to match the examiner’s configuration.

### The virtual multiple errands test (VMET)

**Figure 1:**
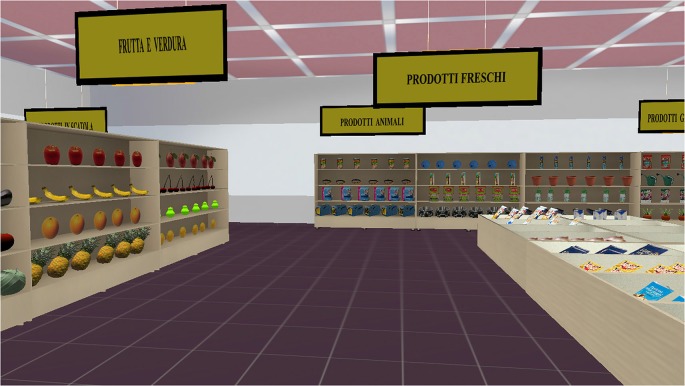
**Screenshot of the virtual multiple errands test (VMET)**.

The VMET consists of a Blender-based application that enables the active exploration of a virtual supermarket, where participants are requested to select and buy various products presented on shelves. From a technical point of view, the VMET was created with the software NeuroVR^1^ (Riva et al., 2011), a free virtual-reality platform for creating virtual environments useful for neuropsychological assessment and neurorehabilitation. NeuroVR is software that allows nonexpert users to adapt the content of several virtual environments to the specific needs of the clinical and research setting. Thanks to the NeuroVR Player, it is possible to visualize virtual environments: The user enters the virtual supermarket, and he/she is presented with virtual objects of the various items to be purchased. Each virtual object has been inserted through the NeuroVR Editor, which offers a rich database of 2D and 3D objects; these can be easily placed into the predesigned virtual scenario by using an icon-based interface. Using a joystick, the participant is able to freely navigate the various aisles (using the up-down joystick arrows) and to collect products (by pressing a button placed on the right side of the joystick), after having selected them with the viewfinder. After an initial training phase with a smaller supermarket, the user enters the virtual supermarket and is presented with virtual objects of the various items to be purchased (Figure 1).

The virtual supermarket contains products grouped into the main grocery categories, including beverages, fruits and vegetables, breakfast foods, hygiene products, frozen foods, garden products, and pet products. Signs at the top of each section indicate the product categories as an aid for navigation.

Participants are also given a shopping list, a map of the supermarket, some information about the supermarket (opening and closing times, products on sale, etc.), a pen, a wrist watch, and the instruction sheet. The instructions are fully illustrated for the participants, and the rules are explained with precise reference to the instruction sheet. The VMET test is composed of four main tasks. The first involves purchasing six items (e.g., one product on sale). The second involves asking the examiner information about one item to be purchased. The third involves writing the shopping list 5 min after beginning the test. The fourth involves responding to some questions at the end of the virtual session by using useful materials (e.g., the closing time of the virtual supermarket). To complete the task, participants have to follow several rules: (1) they have to execute all the proposed tasks; (2) they can execute all the tasks in any order; (3) they cannot go to a place unless it is a part of a task; (4) they cannot pass through the same passage more than once; (5) they cannot buy more than two items per category (look at the chart); (6) they have to take as little time as possible to complete the exercise; (7) they cannot talk to the researcher unless this is a part of the task; and (8) they have to go to their “shopping cart” after 5 min from the beginning of the task and make a list of all their products. The time is stopped when the participant says, “I finished.” During the task, the examiner takes notes on the participant’s behaviors in the virtual environment. As suggested by Shallice and Burgess (1991), the following errors were recorded (please also see the VMET validation procedure in Raspelli et al., 2012): task failures, inefficiencies, strategies, rule breaks, and interpretation failures. A task failure occurs when a subtask is not completed satisfactorily; for example, the first task required participants to purchase six items, so it was composed of six subtasks. For errors in executing the tasks, the scoring range was from 11 (the participants had correctly done the 11 subtasks) to 33 (the participants had totally omitted the 11 subtasks). The scoring scale for each task failure was from 1 to 3 (1 = the participant performed the task 100% correctly as indicated by the test; 2 = the participant performed aspects of the task, but not completed 100% accurately; 3 = the participant totally omitted the task). An inefficiency occurs when a more effective strategy could have been applied to accomplish the task. An example of the eight inefficiencies is not grouping similar tasks when it is possible. The scoring range was from 8 (many inefficiencies) to 32 (no inefficiencies). More precisely, the scoring scale for each inefficiency was from 1 to 4 (1 = always; 2 = more than once; 3 = once; 4 = never). To measure the participant’s ability to use effective strategies that facilitate carrying out the tasks, it is possible to evaluate 13 possible strategies. An example of a good strategy is doing accurate planning before starting a specific subtask. For each strategy, the scoring scale for each strategy was from 1 to 4 (1 = always; 2 = more than once; 3 = once; 4 = never). The total score range was from 13 (good strategies) to 52 (no strategies). A rule break occurs when one of the eight rules listed in the instruction sheet has been violated (e.g., talking with the examiner when not necessary). The scoring scale for each rule break was from 1 to 4 (1 = always; 2 = more than once; 3 = once; 4 = never). For rule breaks, the scoring range was from 8 (a large number of rule breaks) to 32 (no rule breaks). Finally, an interpretation failure occurs when the requirements of a particular task are misunderstood; for example, when a participant thinks that the subtasks all have to be done in the order presented in the information sheet. The scoring range was from 3 (a large number of interpretation failures) to 6 (no interpretation failures). The scoring scale for each interpretation failure was from 1 to 2 (1 = yes; 2 = no).

## Procedure

After participants gave written informed consent to participate, they underwent a neuropsychological global assessment; this was done in order to obtain an accurate overview of their cognitive function and to split the PD sample according to the guidelines of the Task Force for the diagnosis of PD-MCI (Litvan et al., 2012). Then, all participants were required to complete the neuropsychological functions evaluation. At the beginning of the experimental session, participants were asked to sit at a desk in front of a computer monitor to complete the VMET. The VMET was rendered using a portable computer (Intel Core 2 Duo with graphics board OpenGL compatible and 256 MB video memory; operative System was Microsoft Windows XP). Participants also had a gamepad (Logitech Rumble F510), which allowed them to explore and interact with the environment. Then they were asked to complete the VMET procedure after a training session. A training period of about 15 min was first provided in a smaller version of the virtual supermarket environment in order to familiarize participants with the navigation and shopping tasks.

## Results

Data were entered into Microsoft Excel and analyzed using SPSS version 18 (Statistical Package for the Social Sciences–SPSS for Windows, Chicago, IL, USA). To investigate differences in EFs and VMET scores between groups (CG vs. PD-NC vs. PD-MCI), a series of analysis of variance were calculated. *Post hoc* tests (with Bonferroni’s adjustment) were carried out to compare significant differences. The level of significance was set at *α* = 0.05.

### Executive function scores

In order to investigate differences in neuropsychological evaluation of EFs, a series of analysis of variance were computed with *groups* (CG vs. PD-NC vs. PD-MCI) as between variable. Five participants (three of the PD-MCI and two of the PD-NC group) were not included in the Clock Drawing Test analyses for errors in the score recording. Moreover, one patient from the PD-MCI group was excluded from the analyses of the phonological and semantic verbal fluency tests.

Regarding the Clock Drawing Test, results showed significant differences between groups [*F*_(2,37)_ = 9.82, *p* < 0.001, *η_p_*^2^ = 0.347]. In particular, *post hoc* comparisons indicated that PD-MCI patients performed significantly poorer (*M* = 7.62, *SD* = 2.25) when compared with the CG (*M* = 9.83, *SD* = 0.224, *p* < 0.001) and with the PD-NC group (*M* = 9.3, *SD* = 0.804, *p* < 0.01).

In regard to the Phonological verbal fluency test, findings showed significant differences between groups [*F*_(2,41)_ = 34.7, *p* < 0.001, *η_p_*^2^ = 0.629]. *Post hoc* comparisons demonstrated that the CG performed significantly better (*M* = 50.1, *SD* = 8.55) when compared with the PD-MCI (*M* = 22.7, *SD* = 10.1, *p* < 0.001) and PD-NC group (*M* = 32.9, *SD* = 8.55, *p* < 0.001). Moreover, mean scores of the PD-NC group were significantly higher (*p* < 0.05) when compared with those of the PD-MCI group.

In regard to the Semantic verbal fluency test, the one-way ANOVA showed significant differences between groups [*F*_(2,41)_ = 21.8, *p* < 0.001, *η_p_*^2^ = 0.516]. In particular, *post hoc* comparisons revealed that the CG (*M* = 53.4, *SD* = 7.8) performed significantly better when compared with the PD-NC (*M* = 43.5, *SD* = 9.2, *p* < 0.01) and the PD-MCI group (*M* = 33.2, *SD* = 8.01, *p* < 0.001). More interestingly, findings showed that the PD-MCI group was significantly worse (*p* < 0.01) than the PD-NC group.

Finally, analysis conducted on the Tower of London test revealed significant differences between groups [*F*_(2,42)_ = 16.5, *p* < 0.001, *η_p_*^2^ = 0.441]. *Post hoc* comparisons showed that the PD-MCI group performed significantly poorer (*M* = 15.5, *SD* = 5.01) than the CG (*M* = 26.4, *SD* = 4.64, *p* < 0.001) and the PD-NC group (*M* = 23, *SD* = 5.01, *p* < 0.001).

Table 3 summarized the main results.

**Table 3:** **One-way ANOVA results of mean scores obtained by participants divided into the three groups at the EF tasks**.

EF test	Group						*Post-hoc* comparisons		
	PD-NC	PD-MCI	CG	*F*	*p*	$\eta_{p}^{2}$	PD-NC vs. CG	PD-NC vs. PD-MCI	CG vs. PD-MCI
Clock drawing test	9.3 (0.8)	7.6 (2.2)	9.8 (0.2)	9.8	***	0.347	N.S.	**	***
Phonological verbal fluency	32.9 (8.5)	22.7 (10.1)	50.1 (8.5)	34.7	***	0.629	***	*	***
Semantic verbal fluency	43.5 (9.2)	33.2 (8.0)	53.4 (7.8)	21.8	***	0.516	**	**	***
Tower of London test	23 (5.0)	15.5 (5.0)	26.4 (4.6)	16.5	***	0.441	N.S.	***	***

*Values are shown as mean (SD)*.

p values: *** <0.001, ** <0.01, * <0.05, N.S. = Nonsignificant.

### VMET Scores

In order to investigate differences in VMET scores, a series of analysis of variance were computed with *groups* (CG vs. PD-NC vs. PD-MCI) as between variable. First of all, in regard to the time needed for each participant to complete the task, analysis showed significant differences between groups [*F*_(2,42)_ = 3.83, *p* < 0.05, *η_p_*^2^ = 0.154]. In particular, *post hoc* analyses indicated that the PD-MCI group took significantly less time (*M* = 1223, *SD* = 579, *p* < 0.05) compared with CG (*M* = 727, *SD* = 308).

Concerning the task failure, results showed significant differences between groups [*F*_(2,42)_ = 20.2, *p* < 0.001, *η_p_*^2^ = 0.491]. In particular, *post hoc* comparisons indicated that the CG performed significantly better (*M* = 14.3, *SD* = 2.32) when compared with the PD-NC group (*M* = 22.3, *SD* = 4.25, *p* < 0.001) and the PD-MCI group (*M* = 22.8, SD = 5.22, *p* < 0.001).

Regarding inefficiencies, findings revealed significant differences between groups [*F*_(2,42)_ = 3.58, *p* < 0.05, *η_p_*^2^ = 0.146]. *Post hoc* comparisons indicated that the PD-MCI group performed significantly worse (*M* = 18.6, *SD* = 4.03, *p* < 0.05) with respect to the CG (*M* = 24.2, *SD* = 8.18).

Results also showed significant differences between groups in the strategies [*F*_(2,42)_ = 9.82, *p* < 0.001, *η_p_*^2^ = 0.319]. In particular, *post hoc* comparisons indicated that the CG used significantly more effective strategies (*M* = 32.2, *SD* = 5.3) when compared with the PD-NC group (*M* = 40.5, *SD* = 8.69, *p* < 0.01) and the PD-MCI group (*M* = 43.6, *SD* = 7.42, *p* < 0.001).

Finally, no significant differences between groups were found in the rule breaks and in the interpretation failure. Results are summarized in Table 4.

**Table 4:** **One-way ANOVA results of mean scores obtained by participants divided into the three groups at the VMET**.

VMET scores	Group						*Post-hoc* comparisons		
	PD-NC	PD-MCI	CG	*F*	*p*	$\eta_{p}^{2}$	PD-NC vs. CG	PD-NC vs. PD-MCI	CG vs. PD-MCI
Time (seconds)	1110 (600)	1223 (579)	727 (308)	3.8	*	0.347	N.S.	N.S.	*
Task failure	22.3 (4.2)	22.8 (5.2)	14.3 (2.3)	20.2	***	0.491	***	N.S.	***
Inefficiencies	22.3 (4.3)	18.6 (4.0)	24.2 (8.1)	3.6	*	0.146	N.S.	N.S.	*
Strategies	40.5 (8.7)	43.6 (7.4)	32.2 (5.3)	9.8	***	0.319	**	N.S.	***
Rule breaks	23.2 (7.0)	21.9 (5.1)	23.6 (7.0)	0.324	N.S	0.015	N.S.	N.S.	N.S
Interpretation failures	5 (0.8)	5.07 (1.0)	4.47 (0.8)	1.9	N.S	0.086	N.S.	N.S.	N.S.

*Values are shown as mean (SD)*.

p values: *** <0.001, ** <0.01, * <0.05, N.S. = Nonsignificant.

## Discussion and conclusion

Because cognitive impairment is a common complication of PD and is associated with significant disability for patients and a burden for caregivers, it is crucial to fully investigate the distinguishing features of the neuropsychological profile in this clinical population (Aarsland et al., 1999, 2000; Schrag et al., 2000). As the PD progresses, a relevant proportion of patients will develop dementia (Aarsland et al., 2003; Bosboom et al., 2004; Hely et al., 2008). Specifically, Aarsland et al. (2005) found that more than 30% of PD patients have dementia. So the focus now is to identify patients with a potentially higher risk of dementia, with the possibility to implement an early and individualized cognitive rehabilitation treatment to improve their quality of life. Particularly, an increasing number of studies have suggested that the executive deficits in PD are predictive of the conversion to dementia (Levy et al., 2002; Woods and Tröster, 2003).

On these premises, the main objective of this study was to investigate the potentiality of the VMET, to integrate the traditional neuropsychological evaluation of EFs in PD with a more ecologically valid evaluation. This study offers initial evidence that a more ecologically valid evaluation of EFs is more likely to lead to detection of subtle executive deficits in PD patients. VMET specifically seems to capture the early executive dysfunctions of PD-NC patients, while they did not differ in the traditional assessment of EFs when compared to CG.

First, although some recent reviews suggested that executive deficits are present in the early stage of PD (Kudlicka et al., 2011; Ceravolo et al., 2012), our results showed that PD-NC patients were not impaired in the traditional neuropsychological evaluation of EFs when compared with the CG. In fact, in their review, Kudlicka et al. (2011) underlined that studies on EFs in PD are marked by a general lack of clarity in regard to the measure selection and their clinical interpretation. Obviously, it is crucial to acknowledge the possibility that different results across studies might reflect the different tests used, and the underlying functions that the tests are thought to capture. So it is crucial to fully understand which subcomponents of EFs are impaired early in this population. In this direction, Kudlicka et al. (2013) used a data-driven approach to investigate which areas of EF are particularly deficient in 34 patients with PD. Results showed that the impairment was more profound in tests requiring time-efficient attentional control; for example, the Trail Making Test (Tombaugh, 2004).

Our findings showed only a significant difference in the semantic verbal and phonetic verbal fluencies between PD-NC and cognitively healthy participants. As previously explained, verbal fluency tests measure several EF components, including set-switching, strategy generation, and rule attainment, along with other non-EF components such as semantic memory and verbal lexicon. Our results are consistent with a recent meta-analysis that reports verbal fluency deficits in PD (Henry and Crawford, 2004). Specifically, Henry and Crawford (2004) found that PD patients were significantly more impaired in semantic fluency, concluding that this deficit may be associated not only with a problem in executive functioning, but also properly with an initial disorder in the semantic memory (namely, concept-based knowledge). Also, in an interesting study with 88 PD patients and 65 healthy participants, Koerts et al. (2013) pointed out that verbal fluency deficits can be interpreted in light of the progression of the disease and the dysfunctions in other cognitive domains. The performance in the verbal fluency tests is explained by the psychomotor speed in the mild stage of PD, while the cognitive flexibility accounts for deficits in those tests in the moderate phases of the disease.

Concerning the VMET, as previously indicated, our main findings revealed significant differences in some VMET scores between the PD-NC and the cognitively healthy participants. Specifically, within all VMET scores, it is interesting to note a significant difference in task failure and strategies between these two groups. PD-ND patients, compared with cognitively healthy controls, made a greater number of errors in completing the subtasks of VMET. Furthermore, compared with the CG, PD-ND patients showed poorer ability in using effective strategies that facilitate the carrying out of the tasks; for example, accurate planning before starting a specific subtask or using the map for navigating the virtual supermarket. These executive deficits may reflect a specific deficit in cognitive flexibility; namely, the ability with which a person’s conceptualization changes selectively to effectively respond to external/internal stimulation. This may also explain why there is no significant difference between PD-ND and PD-MCI in these VMET scores. Indeed, to discriminate between PD-MCI and PD-ND, it is important to follow the recent guidelines of the Task Force (Litvan et al., 2012). So our findings confirm that the traditional assessment of EFs appears to be more useful to detect differences in the EFs between these two cognitive groups.

In conclusion, our results showed that the VMET appears sensitive to evaluate the functional status of PD with normal cognition, as manifested in terms of executive deficits, which had not been fully acknowledged by traditional neuropsychological evaluations. The VMET allows the possibility to evaluate some subcomponents of EFs in ecological settings, giving a more accurate estimate of the patients’ deficits that are difficult to detect with traditional tests.

As previously explained, one of the most crucial criticisms of the neuropsychological tests is the lack of ecological validity (Goldstein, 1996; Chaytor and Schmitter-Edgecombe, 2003; Chan et al., 2008). Even though patients with supposed executive deficits may perform as well as controls on traditional neuropsychological tests, they may experience difficulties in real world situations. VR may be used to offer a new human-computer interaction paradigm in which patients are active participants within an ecological virtual world (Riva, 2009). In virtual tasks such as the VMET, it is possible to simulate life-like challenges, which require a more complex series of goals to achieve and the cognitive flexibility to elaborate different strategies to accomplish them and to inhibit inappropriate actions.

Our results may also represent a theoretical contribution in the attempt to isolate the specific subcomponents of EF. Most of the traditional neuropsychological tests, therefore, measure one specific EF component, but they don’t reflect a true picture of a functional patient’s status. According to different theories, however, EF is best conceptualized as a system of interconnected processes guided necessarily by a central supervisor system to facilitate goal-oriented behavior (Luria, 1966; Norman and Shallice, 1986; Miller and Cohen, 2001; Miller et al., 2002). Our findings contribute to emphasize the idea that a breakdown in the executive control mechanisms is reflected in deficits in many multitasking behaviors, such as effective planning and strategy allocation and monitoring.

The findings of this study are interesting and valuable, but there are some limitations. First, the small sample size of 45 participants may limit the generalizability of the results. The sample, however, was carefully evaluated with a comprehensive neuropsychological assessment according to the criteria established by Litvan et al. (2012). Second, considering the use of computerized tests for PD patients with motor deficits, it would be important to also assess the individual’s perception of VMET usability (for example, difficulties during the experience in using the joystick, selecting products from aisles, and learning to move in the supermarket). As explained above, a recent study showed good usability of this virtual instrument (Pedroli et al., 2013). The performance on the VMET, however, must be read with consideration of the motor deficit. A final limitation of our study is the difference between the PD and CGs in terms of years of educations. All scores obtained from neuropsychological tests were corrected for education level according to Italian normative data, but the results from VMET must be viewed according to this potential limit. A future challenge is to explore the relative impact of age, gender, education on VMET scores: for example, in an interesting work of Boone (1999) it was found that the impact of educational level and gender was limited to some Wisconsin Card Sorting Test score. Obviously, further studies are needed to evaluate the potentiality of the VMET, especially in terms of its temporal stability, namely, test–retest reliability and criterion validity for PD. This study, however, provides initial evidence that a more ecological evaluation of EFs may provide the possibility to also detect subtle executive deficits in PD-ND patients.

All participants’ data were memorized in encrypted and password-protected files, following the criteria to protect personal health information (El Emam et al., 2011) and using PsychoPass method (Cipresso et al., 2012) to generate and share passwords information among colleagues.

## Authors’ contribution

Conceived and designed the experiments: Pietro Cipresso, Giovanni Albani, Silvia Serino, Alessandro Mauro, Giuseppe Riva. Performed the experiments: Elisa Pedroli. Analyzed the data: Pietro Cipresso, Silvia Serino, Federica Pallavicini. Wrote the first version of the paper: Silvia Serino. Revised and contributed to the last version of the paper: Pietro Cipresso, Giovanni Albani, Silvia Serino, Elisa Pedroli, Federica Pallavicini, Alessandro Mauro, Giuseppe Riva.

## Conflict of interest statement

The authors declare that the research was conducted in the absence of any commercial or financial relationships that could be construed as a potential conflict of interest.

## Footnotes

^1^www.neurovr.org

## References

[B1] Aarsland D. Andersen K. Larsen J. P. Lolk A. Kragh-Sørensen P. (2003). Prevalence and characteristics of dementia in Parkinson disease: an 8-year prospective study. Arch. Neurol. 60, 387–392. 10.1001/archneur.60.3.387 12633150

[B2] Aarsland D. Brønnick K. Fladby T. (2011). Mild cognitive impairment in Parkinson’s disease. Curr. Neurol. Neurosci. Rep. 11, 371–378. 10.1007/s11910-011-0203-1 21487730

[B3] Aarsland D. Kvaløy J. T. Andersen K. Larsen J. P. Tang M. X. Lolk A.. (2007). The effect of age of onset of PD on risk of dementia. J. Neurol. 254, 38–45. 10.1007/s00415-006-0234-8 17508138

[B4] Aarsland D. Larsen J. P. Karlsen K. Lim N. G. Tandberg E. (1999). Mental symptoms in Parkinson’s disease are important contributors to caregiver distress. Int. J. Geriatr. Psychiatry 14, 866–874. 10.1002/(SICI)1099-1166(199910)14:10<866::AID-GPS38=3.0.CO;2-Z 10521886

[B5] Aarsland D. Larsen J. P. Tandberg E. Laake K. (2000). Predictors of nursing home placement in Parkinson’s disease: a population-based, prospective study. J. Am. Geriatr. Soc. 48, 938–942. 10.1002/gps.930080906 10968298

[B6] Aarsland D. Zaccai J. Brayne C. (2005). A systematic review of prevalence studies of dementia in Parkinson’s disease. Mov. Disord. 20, 1255–1263. 10.1002/mds.20527 16041803

[B7] Albani G. Raspelli S. Carelli L. Priano L. Pignatti R. Morganti F.. (2011). Sleep dysfunctions influence decision making in undemented Parkinson’s disease patients: a study in a virtual supermarket. Stud. Health Technol. Inform. 163, 8–10. 10.3233/978-1-60750-706-2-8 21335749

[B99] Alderman N. Burgess P. W. Knight C. Henman C. (2003). Ecological validity of a simplified version of the Multiple Errands Shopping Test. J. Int. Neuropsychol. Soc. 9, 31–44. 10.1017/s1355617703910046 12570356

[B8] Barker L. A. Andrade J. Morton N. Romanowski C. A. J. Bowles D. P. (2010). Investigating the ‘latent’deficit hypothesis: age at time of head injury, implicit and executive functions and behavioral insight. Neuropsychologia 48, 2550–2563. 10.1016/j.neuropsychologia.2010.05.001 20470806

[B9] Barker L. A. Morton N. Morrison T. G. McGuire B. E. (2011). Inter-rater reliability of the Dysexecutive Questionnaire (DEX): comparative data from non-clinician respondents-all raters are not equal. Brain Inj. 25, 997–1004. 10.3109/02699052.2011.597046 21749190

[B10] Bechara A. Damasio A. R. Damasio H. Anderson S. W. (1994). Insensitivity to future consequences following damage to human prefrontal cortex. Cognition 50, 7–15. 10.1016/0010-0277(94)90018-3 8039375

[B98] Biundo R. Calabrese M. Weis L. Facchini S. Ricchieri G. Gallo P.. (2013). Anatomical correlates of cognitive functions in early Parkinson’s disease patients. PLoS One 8:e64222. 10.1371/journal.pone.0064222 23717572 PMC3661458

[B11] Bohil C. J. Alicea B. Biocca F. A. (2011). Virtual reality in neuroscience research and therapy. Nat. Rev. Neurosci. 12, 752–762. 10.1038/nrn3122 22048061

[B12] Boone K. B. (1999). “Neuropsychological assessment of executive functions: impact of age, education, gender, intellectual level and vascular status on executive test scores,” in The human frontal lobes: Functions and disorders. The science and practice of neuropsychology series, eds Miller L. B. Cummings J. L. (New York, NY, US: Guilford Press), 247–260.

[B13] Bosboom J. L. W. Stoffers D. Wolters E. C. (2004). Cognitive dysfunction and dementia in Parkinson’s disease. J. Neural Transm. 111, 1303–1315 10.1007/s00702-004-0168-1 15480840

[B14] Burgess P. W. Alderman N. Forbes C. Costello A. Ma Coates L. Dawson D. R.. (2006). The case for the development and use of “ecologically valid” measures of executive function in experimental and clinical neuropsychology. J. Int. Neuropsychol. Soc. 12, 194–209. 10.1017/S1355617706060310 16573854

[B15] Burgess P. W. Shallice T. (1996a). Bizarre responses, rule detection and frontal lobe lesions. Cortex 32, 241–259. 10.1016/s0010-9452(96)80049-9 8800613

[B16] Burgess P. W. Shallice T. (1996b). Response suppression, initiation and strategy use following frontal lobe lesions. Neuropsychologia 34, 263–272. 10.1016/0028-3932(95)00104-2 8657357

[B17] Burgess P. W. Simons J. S. (2005). “Theories of frontal lobe executive function: clinical applications,” in Effectiveness of Rehabilitation for Cognitive Deficits, eds Halligan P. W. Wade D. T. (New York: Oxford University Press), 211–231.

[B18] Burgess P. W. Veitch E. de Lacy Costello A. Shallice T. (2000). The cognitive and neuroanatomical correlates of multitasking. Neuropsychologia 38, 848–863. 10.1016/S0028-3932(99)00134-7 10689059

[B19] Buter T. C. Van den Hout A. Matthews F. E. Larsen J. P. Brayne C. Aarsland D. (2008). Dementia and survival in Parkinson disease a 12-year population study. Neurology 70, 1017–1022. 10.1212/01.wnl.0000306632.43729.24 18362281

[B20] Caffarra P. Gardini S. Zonato F. Concari L. Dieci F. Copelli S.. (2011). Italian norms for the Freedman version of the clock drawing test. J. Clin. Exp. Neuropsychol. 33, 982–988. 10.1080/13803395.2011.589373 22082081

[B21] Ceravolo R. Pagni C. Tognoni G. Bonuccelli U. (2012). The epidemiology and clinical manifestations of dysexecutive syndrome in Parkinson’s disease. Front. Neurol. 3:159. 10.3389/fneur.2012.00159 23162529 PMC3497716

[B22] Chan R. C. K. Shum D. Toulopoulou T. Chen E. Y. H. (2008). Assessment of executive functions: review of instruments and identification of critical issues. Arch. Clin. Neuropsychol. 23, 201–216. 10.1016/j.acn.2007.08.010 18096360

[B23] Chaytor N. Schmitter-Edgecombe M. (2003). The ecological validity of neuropsychological tests: a review of the literature on everyday cognitive skills. Neuropsychol. Rev. 13, 181–197. 10.1023/B:NERV.0000009483.91468.fb 15000225

[B103] Cipresso P. Gaggioli A. Serino S. Cipresso S. Riva G. (2012). How to create memorizable and strong passwords. J. Med. Internet Res. 14, e10. 10.2196/jmir.1906 22233980 PMC3846346

[B24] Cipresso P. La Paglia F. La Cascia C. Riva G. Albani G. La Barbera D. (2013a). Break in volition: a virtual reality study in patients with obsessive-compulsive disorder. Exp. Brain Res. 229, 443–449. 10.1007/s00221-013-3471-y 23535833

[B25] Cipresso P. Serino S. Pedroli E. Albani G. Riva G. (2013b). “Psychometric reliability of the neuroVR-based virtual version of the multiple errands test,” in 7th International Conference on Pervasive Computing Technologies for Healthcare (Venice, Italy). 10.4108/icst.pervasivehealth.2013.252361

[B26] Cole M. W. Laurent P. Stocco A. (2013). Rapid instructed task learning: a new window into the human brain’s unique capacity for flexible cognitive control. Cogn. Affect. Behav. Neurosci. 13, 1–22. 10.3758/s13415-012-0125-7 23065743 PMC3557598

[B27] Cools R. Barker R. A. Sahakian B. J. Robbins T. W. (2001). Mechanisms of cognitive set flexibility in Parkinson’s disease. Brain 124, 2503–2512. 10.1093/brain/124.12.2503 11701603

[B28] Cools R. Clark L. Robbins T. W. (2004). Differential responses in human striatum and prefrontal cortex to changes in object and rule relevance. J. Neurosci. 24, 1129–1135. 10.1523/JNEUROSCI.4312-03.2004 14762131 PMC6793591

[B29] Cools A. R. van den Bercken J. H. Horstink M. W. van Spaendonck K. P. Berger H. J. (1984). Cognitive and motor shifting aptitude disorder in Parkinson’s disease. J. Neurol. Neurosurg. Psychiatry 47, 443–453. 10.1136/jnnp.47.5.443 6736974 PMC1027818

[B31] Damasio A. R. (1995). Toward a neurobiology of emotion and feeling: operational concepts and hypotheses. Neuroscientist 1, 19–25 10.1177/107385849500100104

[B32] Dreher J. C. Koechlin E. Tierney M. Grafman J. (2008). Damage to the fronto-polar cortex is associated with impaired multitasking. PLoS One 3:e3227. 10.1371/journal.pone.0003227 18795100 PMC2528949

[B102] El Emam K. Moreau K. Jonker E. (2011). How strong are passwords used to protect personal health information in clinical trials? J. Med. Internet Res. 13, 13–22. 10.2196/jmir.1335 21317106 PMC3221339

[B33] Fancello S. G. Vio C. Cianchetti C. (2006). Torre di Londra, Test di Valutazione delle Funzioni Esecutive (Pianificazione e Problem Solving) [Tower of London, a Test for Executive Functions (Planning and Problem Solving)]. Trento: Edizioni Centro Studi Erickson.

[B34] Folstein M. F. Folstein S. E. McHugh P. R. (1975). “Mini-mental state”. A practical method for grading the cognitive state of patients for the clinician. J. Psychiatr. Res. 12, 189–198. 10.1016/0022-3956(75)90026-6 1202204

[B35] Foltynie T. Brayne C. E. G. Robbins T. W. Barker R. A. (2004). The cognitive ability of an incident cohort of Parkinson’s patients in the UK. The CamPaIGN study. Brain 127, 550–560. 10.1093/brain/awh067 14691062

[B36] Freedman M. Leach L. Kaplan E. Winocur G. Shulman K. I. Delis D. C. (1994). Clock Drawing: a Neuropsychological Analysis. Oxford: Oxford University Press.

[B37] Girotti F. Soliveri P. Carella F. Piccolo I. Caffarra P. Musicco M.. (1988). Dementia and cognitive impairment in Parkinson’s disease. J. Neurol. Neurosurg. Psychiatry 51, 1498–1502. 10.1136/jnnp.51.12.1498 3221216 PMC1032763

[B38] Goldberg E. (2009). The New Executive Brain: Frontal Lobes in a Complex World. Oxford: Oxford University Press.

[B39] Goldstein G. (1996). “Functional considerations in neuropsychology,” in Ecological Validity of Neuropsychological Testing, eds Sbordone R. J. Long C. J. (Delray Beach, FL: GR Press/St. Lucie Press), 75–89.

[B40] Grafman J. Litvan I. (1999). Importance of deficits in executive functions. Lancet 354, 1921–1923. 10.1016/S0140-6736(99)90438-5 10622291

[B41] Greenlief C. L. Margolis R. B. Erker G. J. (1985). Application of the trail making test in differentiating neuropsychological impairment of elderly persons. Percept. Mot. Skills 61, 1283–1289. 10.2466/pms.1985.61.3f.1283 4094872

[B42] Hely M. A. Reid W. G. J. Adena M. A. Halliday G. M. Morris J. G. L. (2008). The Sydney multicenter study of Parkinson’s disease: the inevitability of dementia at 20 years. Mov. Disord. 23, 837–844. 10.1002/mds.21956 18307261

[B43] Henry J. D. Crawford J. R. (2004). Verbal fluency deficits in Parkinson’s disease: a meta-analysis. J. Int. Neuropsychol. Soc. 10, 608–622. 10.1017/S1355617704104141 15327739

[B44] Hikosaka O. Isoda M. (2010). Switching from automatic to controlled behavior: cortico-basal ganglia mechanisms. Trends Cogn. Sci. 14, 154–161. 10.1016/j.tics.2010.01.006 20181509 PMC2847883

[B45] Jacobs D. M. Marder K. Côté L. J. Sano M. Stern Y. Mayeux R. (1995). Neuropsychological characteristics of preclinical dementia in Parkinson’s disease. Neurology 45, 1691–1696. 10.1212/WNL.45.9.1691 7675228

[B46] Janvin C. C. Larsen J. P. Aarsland D. Hugdahl K. (2006). Subtypes of mild cognitive impairment in Parkinson’s disease: progression to dementia. Mov. Disord. 21, 1343–1349. 10.1002/mds.20974 16721732

[B47] Kehagia A. A. Barker R. A. Robbins T. W. (2010). Neuropsychological and clinical heterogeneity of cognitive impairment and dementia in patients with Parkinson’s disease. Lancet Neurol. 9, 1200–1213. 10.1016/S1474-4422(10)70212-X 20880750

[B48] Kemps E. Szmalec A. Vandierendonck A. Crevits L. (2005). Visuo-spatial processing in Parkinson’s disease: evidence for diminished visuo-spatial sketch pad and central executive resources. Parkinsonism Relat. Disord. 11, 181–186. 10.1016/j.parkreldis.2004.10.010 15823483

[B101] Koerts J. Meijer H. A. Colman K. S. F. Tucha L. Lange K. W. Tucha O. (2013). What is measured with verbal fluency tests in Parkinson’s disease patients at different stages of the disease? J. Neural Transm. 120, 403–411. 10.1007/s00702-012-0885-9 22922998

[B49] Kudlicka A. Clare L. Hindle J. V. (2011). Executive functions in Parkinson’s disease: systematic review and meta-analysis. Mov. Disord. 26, 2305–2315. 10.1002/mds.23868 21971697

[B50] Kudlicka A. Clare L. Hindle J. V. (2013). Pattern of executive impairment in mild to moderate parkinson’s disease. Dement. Geriatr. Cogn. Disord. 36, 50–66. 10.1159/000348355 23774679

[B51] Leber A. B. Turk-Browne N. B. Chun M. M. (2008). Neural predictors of moment-to-moment fluctuations in cognitive flexibility. Proc. Natl. Acad. Sci. U S A 105, 13592–13597. 10.1073/pnas.0805423105 18757744 PMC2527350

[B52] Levy G. Jacobs D. M. Tang M. X. Côté L. J. Louis E. D. Alfaro B.. (2002). Memory and executive function impairment predict dementia in Parkinson’s disease. Mov. Disord. 17, 1221–1226. 10.1002/mds.10280 12465060

[B53] Lezak M. D. (1995). Neuropsychological Assessment. Oxford: Oxford University Press.

[B54] Litvan I. Goldman J. G. Tröster A. I. Schmand B. A. Weintraub D. Petersen R. C.. (2012). Diagnostic criteria for mild cognitive impairment in Parkinson’s disease: movement disorder society task force guidelines. Mov. Disord. 27, 349–356. 10.1002/mds.24893 22275317 PMC3641655

[B55] Luria A. R. (1966). Higher Cortical Functions in Man. New York: Basic Books.

[B56] Magni E. Binetti G. Bianchetti A. Rozzini R. Trabucchi M. (1996). Mini-mental state examination: a normative study in Italian elderly population. Eur. J. Neurol. 3, 198–202. 10.1111/j.1468-1331.1996.tb00423.x 21284770

[B57] McKinlay A. Grace R. C. Dalrymple-Alford J. C. Roger D. (2010). Characteristics of executive function impairment in Parkinson’s disease patients without dementia. J. Int. Neuropsychol. Soc. 16, 268–277. 10.1017/S1355617709991299 20003582

[B58] Miller E. K. Cohen J. D. (2001). An integrative theory of prefrontal cortex function. Annu. Rev. Neurosci. 24, 167–202. 10.1146/annurev.neuro.24.1.167 11283309

[B59] Miller E. K. Freedman D. J. Wallis J. D. (2002). The prefrontal cortex: categories, concepts and cognition. Philos. Trans. R. Soc. Lond. B Biol. Sci. 357, 1123–1136. 10.1098/rstb.2002.1099 12217179 PMC1693009

[B60] Mondini S. Mapelli D. Vestri A. Bisiacchi P. (2003). Esame Neuropsicologico Breve. Milan: Raffaello Cortina Editore.

[B61] Montse A. Pere V. Carme J. Francesc V. Eduardo T. (2001). Visuospatial deficits in Parkinson’s disease assessed by judgment of line orientation test: error analyses and practice effects. J. Clin. Exp. Neuropsychol. 23, 592–598. 10.1076/jcen.23.5.592.1248 11778636

[B62] Morton N. Barker L. (2010). The contribution of injury severity, executive and implicit functions to awareness of deficits after traumatic brain injury (TBI). J. Int. Neuropsychol. Soc. 16, 1089–1098. 10.1017/S1355617710000925 20735889

[B63] Muslimović D. Post B. Speelman J. D. Schmand B. (2005). Cognitive profile of patients with newly diagnosed Parkinson disease. Neurology 65, 1239–1245. 10.1212/01.wnl.0000180516.69442.95 16247051

[B64] Nelson H. E. (1976). A modified card sorting test sensitive to frontal lobe defects. Cortex 12, 313–324. 10.1016/S0010-9452(76)80035-4 1009768

[B65] Norman D. A. Shallice T. (1986). “Attention to action: willed and automatic control of behaviour,” in Consciousness and Self-Regulation: Advances in Research and Theory, eds Davidson R. J. Schwartz G. E. Shapiro D. (New York: Plenum), 1–18.

[B66] Novelli G. Papagno C. Capitani E. Laiacona M. (1986a). Tre test clinici di memoria verbale a lungo termine: Taratura su soggetti normali. / three clinical tests for the assessment of verbal long-term memory function: norms from 320 normal subjects. Arch. Psicol. Neurol. Psichiatr. 47, 278–296.

[B67] Novelli G. Papagno C. Capitani E. Laiacona N. Vallar G. Cappa S. F. (1986b). Tre test clinici di ricerca e produzione lessicale. Taratura su soggetti normali. Arch. Psicol. Neurol. Psichiatr. 47, 477–506.

[B68] Orsini A. Grossi D. Capitani E. Laiacona M. Papagno C. Vallar G. (1987). Verbal and spatial immediate memory span: normative data from 1355 adults and 1112 children. Ital. J. Neurol. Sci. 8, 537–548. 10.1007/bf02333660 3429213

[B69] Otero M. L. Barker A. L. (2013). “The frontal lobes and the executive functioning,” in Handbook of Executive Functioning, eds Goldstein S. Naglieri J. A. (German: Springer), 29–44.

[B70] Padovani A. Costanzi C. Gilberti N. Borroni B. (2006). Parkinson’s disease and dementia. Neurol. Sci. 27, S40–S43. 10.1007/s10072-006-0546-6 16708183

[B71] Pagonabarraga J. Kulisevsky J. (2012). Cognitive impairment and dementia in Parkinson’s disease. Neurobiol. Dis. 46, 590–596. 10.1016/j.nbd.2012.03.029 22484304

[B72] Pedroli E. Cipresso P. Serino S. Albani G. Riva G. (2013). “A virtual reality test for the assessment of cognitive deficits: usability and perspectives,” in 7th International Conference on Pervasive Computing Technologies for Healthcare (Venice, Italy).

[B73] Petersen R. C. (2004). Mild cognitive impairment as a diagnostic entity. J. Intern. Med. 256, 183–194. 10.1111/j.1365-2796.2004.01388.x 15324362

[B74] Petersen R. C. Doody R. Kurz A. Mohs R. C. Morris J. C. Rabins P. V.. (2001). Current concepts in mild cognitive impairment. Arch. Neurol. 58, 1985–1992. 10.1001/archneur.58.12.1985 11735772

[B75] Petrova M. Raycheva M. Zhelev Y. Traykov L. (2010). Executive functions deficit in Parkinson’s disease with amnestic mild cognitive impairment. Am. J. Alzheimers Dis. Other Demen. 25, 455–460. 10.1177/1533317510370956 20484747 PMC10845324

[B76] Ramanan S. Kumar D. (2013). Prospective memory in Parkinson’s disease: a meta-analysis. J. Int. Neuropsychol. Soc. 19, 1109–1118. 10.1017/S1355617713001045 24044729

[B77] Raspelli S. Pallavicini F. Carelli L. Morganti F. Pedroli E. Cipresso P. (2012). Validating the neuro VR-based virtual version of the multiple errands test: preliminary results. Presence: Teleoperators and Virtual Environ. 21, 31–42 10.1162/pres_a_00077

[B78] Riva G. (2009). Virtual reality: an experiential tool for clinical psychology. Br. J. Guid. Counc. 37, 337–345 10.1080/03069880902957056

[B100] Riva G. Gaggioli A. Grassi A. Raspelli S. Cipresso P. Pallavicini F.. (2011). NeuroVR 2—a free virtual reality platform for the assessment and treatment in behavioral health care. Stud. Health Technol. Inform. 163, 493–495. 21335845

[B79] Schrag A. Jahanshahi M. Quinn N. (2000). What contributes to quality of life in patients with Parkinson’s disease?. J. Neurol. Neurosurg. Psychiatry 69, 308–312. 10.1136/jnnp.69.3.308 10945804 PMC1737100

[B80] Shallice T. (1982). Specific impairments of planning. Philos. Trans. R. Soc. Lond. B Biol. Sci. 298, 199–209. 10.1098/rstb.1982.0082 6125971

[B81] Shallice T. Burgess P. W. (1991). Deficits in strategy application following frontal lobe damage in man. Brain 114, 727–741. 10.1093/brain/114.2.727 2043945

[B82] Spinnler H. Tognoni G. (1987). Standardizzazione e taratura Italiana di test neuropsicologici. Ital. J. Neurol. Sci. 6(Suppl. 8). 3330072

[B83] Strauss E. Sherman E. M. S. Spreen O. (2006). A Compendium of Neurpsychological Tests. Admnistration, Norms and Commentary. Oxford: Oxford University Press.

[B84] Stroop J. R. (1935). Studies of interference in serial verbal reactions. J. Exp. Psychol. 18, 643–662 10.1037/h0054651

[B85] Stuss D. T. (2007). “New approaches to prefrontal lobe testing,” in The Human Frontal Lobes: Functions and Disorders, eds Miller B. L. Cummings J. L. (New York: The Guilford), 292–305.

[B86] Stuss D. T. Shallice T. Alexander M. P. Picton T. W. (1995). A multidisciplinary approach to anterior attentional functions. Ann. N Y Acad. Sci. 769, 191–212. 10.1111/j.1749-6632.1995.tb38140.x 8595026

[B87] Tekin S. Cummings J. L. (2002). Frontal-subcortical neuronal circuits and clinical neuropsychiatry: an update. J. Psychosom. Res. 53, 647–654. 10.1016/S0022-3999(02)00428-2 12169339

[B88] Tombaugh T. N. (2004). Trail making test A and B: normative data stratified by age and education. Arch. Clin. Neuropsychol. 19, 203–214. 10.1016/s0887-6177(03)00039-8 15010086

[B89] Verleden S. Vingerhoets G. Santens P. (2007). Heterogeneity of cognitive dysfunction in Parkinson’s disease: a cohort study. Eur. Neurol. 58, 34–40. 10.1159/000102164 17483583

[B90] Vingerhoets G. Verleden S. Santens P. Miatton M. De Reuck J. (2003). Predictors of cognitive impairment in advanced Parkinson’s disease. J. Neurol. Neurosurg. Psychiatry 74, 793–796. 10.1136/jnnp.74.6.793 12754355 PMC1738465

[B91] Whittington C. J. Podd J. Stewart-Williams S. (2006). Memory deficits in Parkinson’s disease. J. Clin. Exp. Neuropsychol. 28, 738–754. 10.1080/13803390590954236 16723322

[B92] Williams-Gray C. H. Evans J. R. Goris A. Foltynie T. Ban M. Robbins T. W.. (2009). The distinct cognitive syndromes of Parkinson’s disease: 5 year follow-up of the CamPaIGN cohort. Brain 132, 2958–2969. 10.1093/brain/awp245 19812213

[B93] Wilson B. A. Alderman N. Burgess P. W. Emslie H. Evans J. J. (1996). Behavioural Assessment of the Dysexecutive Syndrome. London: Thames Valley Test Company.

[B96] Wilson B. Cockburn J. Halligan P. W. (1987). The Behavioural Inattention Test. Bury St. Edmunds: Thames Valley Test Company.

[B95] Wilson B. Cockburn J. Halligan P. (2010). Behavioural Inattention Test. Manuale con Taratura Italiana. Firenze: Giunti Organizzazioni Speciali.

[B94] Wilson B. A. Evans J. J. Emslie H. Alderman N. Burgess P. (1998). The development of an ecologically valid test for assessing patients with a dysexecutive syndrome. Neuropsychol. Rehabil. 8, 213–228 10.1080/713755570

[B97] Woods S. P. Tröster A. I. (2003). Prodromal frontal/executive dysfunction predicts incident dementia in Parkinson’s disease. J. Int. Neuropsychol. Soc. 9, 17–24. 10.1017/S1355617703910022 12570354

